# The effects of fluid absorption and plasma volume changes in athletes following consumption of various beverages

**DOI:** 10.1186/s13102-022-00583-2

**Published:** 2022-12-08

**Authors:** Hyo-Jun Yun, Ji-Yong Lee, Minsoo Jeon, Sang-eun Oh, Jae-Hyeon Park, Jiwun Yoon

**Affiliations:** Center for Sports and Performance Analysis, Korea National Sport University, Seoul, Republic of Korea

**Keywords:** Athletes, Oral rehydration solution, Rehydration, Sports drink

## Abstract

**Background:**

To verify the hydration effects of oral rehydration solution (ORS) on athletes by comparing the degrees of fluid absorption and plasma volume changes following beverage consumption, including ORS.

**Methods:**

Thirty-one participants visited the testing laboratory 4 times at 1-week intervals to consume 1 L of beverage (e.g., water, ORS, and two sports drinks [SpD]) for 30 min on each visit. The urine output was measured 4 times at 1 h, 2 h, 3 h, and 4 h after beverage consumption. A blood sample was collected 3 times at 1 h, 2 h, and 3 h after beverage consumption. Body weight was measured once in 4 h after beverage consumption.

**Results:**

Body weight change was smaller for ORS than for water, SpD1, and SpD2 (*p* < 0.05). Cumulative urine output in 4 h was lower for ORS, SpD1, and SpD2 than for water (*p* < 0.05), and it was lower for ORS than for SpD2 (*p* < 0.05). BHI in 4 h was higher for ORS, SpD1, and SpD2 than for water (*p* < 0.05), and it was higher for ORS than for SpD2 (*p* < 0.05). There was no significant difference in PVC for different beverages at all test times, i.e.., 1 h, 2 h, and 3 h.

**Conclusions:**

We evaluated the hydration effects of the consumption of beverages, such as water, SpD, and ORS in athletes. ORS and SpD were more effective than water. A comparison between ORS and SpD showed that the result could vary depending on the type of SpD.

## Background

Excessive water loss during sports training or competition may cause dehydration in athletes. Consequently, exercise performance may decline due to decreased blood pressure, increased heart rate, and reduced cardiac function [21]. Adequate hydration during training or competition prevents dehydration, maintains optimal body temperature, and improves performance [6, 7]. Reports show higher adrenaline levels in athletes following fluid consumption after dehydration than in those without fluid intake [1, 19]. Therefore, the American College of Sports Medicine (ACSM) recommends that effective rehydration requires the intake of a greater volume of fluid (e.g., 125–150%) than the final fluid deficit (eg, 1.25–1.5 L fluid for every 1 kg BW lost [12].

Athletes usually drink water or sports drinks (SpD) to rehydrate during training and competition. This effect has been demonstrated in several studies [10, 15, 16]. Still, one can also hydrate with water alone or with an oral rehydration solution (ORS) containing water with traces of sugar, salt, sodium, and potassium. However, it is difficult to find studies demonstrating the effectiveness of hydration through ORS in athletes.

ORS was introduced by the World Health Organization (WHO) in 1975 for children with diarrhoea due to cholera [17]. The standard solution, as defined by the WHO-ORS, has an osmolarity of 311 mOsm/L, sodium concentration of 90 mEq/L, potassium concentration of 20 mEq/L, chloride concentration of 80 mEq/L, and glucose concentration of 20 g/L.

An advantage of ORS is that it is easier to administer than intravenous fluid therapy (IVT) and does not cause pain or phlebitis [20]. Meta-analyses have shown that the efficacy of ORS is comparable to that of IVT in treating acute gastroenteritis in children [9, 11], with no significant difference between the ORS group and the IVT group for the frequency of bowel movements, duration of diarrhoea, increase in body weight, hyponatraemia, and hypernatraemia.

Sollanek et al.’s study on 40 adults (male and female) found that hydration with ORS was significantly more effective than water, and there were no significant differences between the effects of sports drinks and water consumption. Maughan et al.’s study on 72 adult males revealed that ORS was more effective than water, with no significant differences between sports drinks and water. These results show that hydration with ORS is more effective than water or sports drinks. However, previous studies were conducted on the general population who were not athletes. De Gray’s study [4] was the first to verify the genetic differences between athletes and the general public. It was reported that the two groups tend to be genetically different, thus, their physiologic responses may differ. Here, we aim to evaluate the hydration effects of ORS by comparing fluid absorption and plasma volume changes in athletes following the consumption of various types of beverages.

## Methods

### Participants

We recruited 36 male college athletes who met the following inclusion criteria. No participant competed in sports requiring an excessive level of weight management, such as weight cut, during the experimental period. The participants demonstrated a thorough understanding of the research after receiving an explanation, and they volunteered to participate in the study. They signed an agreement to comply with the guidelines and precautions. 1 participant dropped out of the study, and we excluded 4 participants who exhibited unexpected behaviours (2 participants unable to hold their urine, 2 participants from the national team) during the experiment. Finally, 31 participants were considered for the study [Age (years): 20.1 $\pm$ 1.14, Height (cm): 174.5 $\pm$ 8.40, Body mass (kg): 76.7 $\pm$ 18.09, BMI (kg/m^2^): 24.7 $\pm$ 4.01]. The statistical power (1-β) is 0.999 when the effect size (ES) and the probability of making a type I error (α) are set to ES = 0.5 and α = 0.05, respectively. Statistical power estimation was performed using the G*Power 3 program [8].

### Test beverages

The test beverages were water, ORS (Lingtea Co., Ltd., Seoul, Republic of Korea), Sports Drink 1 (Lingtea Co., Ltd., Seoul, Republic of Korea), and Sports Drink 2 (Otsuka Pharmaceutical Co., Ltd., Tokyo, Japan). The composition of the beverage is presented in Table 1. All drinks were sealed and stored at 4–6˚C until served.

**Table 1:** Beverage composition

Drink	Energy (kcal/L)	Carbohydrate (g/L)	Fat (g/L)	Protein (g/L)	Osmolality (mOsm/L)	Sodium (mg/L)	Potassium (mg/L)
Water	0	0	0	0	2	0	0
ORS	84	18	0.5	1.7	220	1086	5
SpD1	90	23	0	0	297	174	99
SpD2	250	62	0	0	326	490	200

ORS, oral rehydration solution; SpD, sports drink

### Experimental design

This study used a cross-over design to mitigate the errors related to interpersonal differences. We compared the degrees of fluid absorption after a participant consumed four different types of beverages. The participants did not consume multiple beverages in one session. The experiment was conducted once a week over four weeks continuously. We used a counterbalanced measures design to deal with the order effect. We assigned the participants to four groups determined by adaptive randomisation of age, height, and body weight. The experimental design is presented in Table 2.

**Table 2:** Experimental design

	1st	2nd	3rd	4th
Cohort 1	Water	ORS	SpD1	SpD2
Cohort 2	SpD2	Water	ORS	SpD1
Cohort 3	SpD1	SpD2	Water	ORS
Cohort 4	ORS	SpD1	SpD2	Water

ORS, oral rehydration solution; SpD, sports drink

### Experimental procedures

The experimental procedure is as follows. A participant consumed 2 L of drinking water between 6 and 10 pm on the day before the test. On the test day, the participant woke up at 6:30 am and passed urine into a sterile tube. Next, he consumed 50 ml of drinking water from 7 am for 15 min. The participant arrived at the testing lab by 8 am and provided a urine sample, a blood sample, and body weight measurements. Starting at 8:30 am, the participant consumed 1 L of the assigned beverage, 200 ml every 7.5 min. After finishing the beverage, 3 blood collections and 4 urine measurements were made at 1-h intervals. Finally, the body weight was measured. The experimental schedule is presented in Table 3.

**Table 3:** Experimental schedule

Time	Schedule
6:00 pm–10:00 pm	Consume 2 L of drinking water
06:30 am	Pass urine into a sterilised tube
07:00 am	Consume 500 ml of drinking water for 15 min
07:50 am	Arrive at the testing lab
08:00 am	Provide a urine sample, measure body weight, provide a blood sample
08:30 am–09:00 am	Consume 1 L of assigned beverage (250 ml every 7.5 min)
10:00 am	Measurement of urine output; blood sample collection
11:00 am	Measurement of urine output; blood sample collection
12:00 pm	Measurement of urine output; blood sample collection
1:00 pm	Measurement of urine output; measure body weight

### Measurements

Fluid absorption was confirmed by body weight changes, cumulative urine output, beverage hydration index (BHI), and plasma volume change (PVC). The body weight was measured in units of 0.1 kg (Model DB-60H, CAS) and urine output was measured in units of 0.1 g (Model XE-6000, CAS). Haemoglobin (Hb) and haematocrit (Hct) values were obtained from the analysis of a 3 ml blood sample (Model XN 1000, Sysmex). The BHI was urine output after water consumption divided by urine output after beverage consumption [3]. The BHI of water is 1, so > 1 means that the assigned beverage provides more hydration capability than water, while < 1 means poorer hydration than water. The PVC was calculated using the Hb and Hct values in Eq. (1) [5].

1 $$\Delta {\text{PV}},{\text{\% }} = 100{ }(PV_{A} - PV_{B} ){/}PV_{B}$$

$$BV_{A} = BV_{B} \left( {Hb_{B} /Hb_{A} } \right)$$

$$CV_{A} = { }BV_{A} \left( {Hct_{A} } \right)$$

$$PV_{A} = { }BV_{A} - CV_{A}$$

BV, blood volume; CV, red cell volume; PV, plasma volume; Hb, hemoglobin; Hct, haematocrit; B, before drinking beverages; A, after drinking beverages.

### Data and statistical analysis

The Kolmogorov-Smirnova test was performed to confirm the normality of body weight change, cumulative urine output, BHI, and PVC for each drink. The 1-way repeated-measures ANOVA was performed for each beverage to confirm the differences in body weight change, cumulative urine output, BHI, and PVC. The LSD was conducted for the post-hoc test. All statistical analyses were performed using the statistical package for social sciences (SPSS), version 25.0 for Windows. Statistical significance was accepted for < 0.05. Data were presented as means ± SDs.

## Results

### Body mass change

**Fig. 1:**
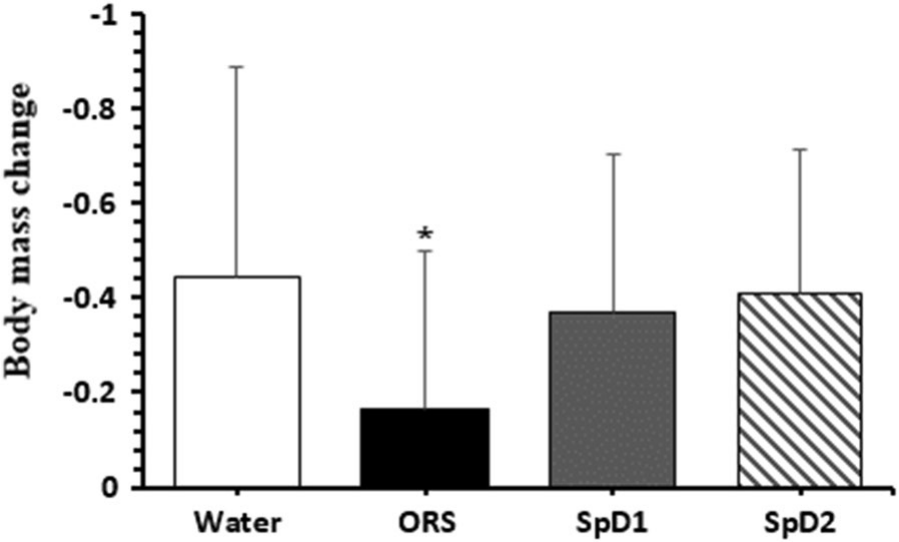
Body mass change following the consumption of test drinks. Values are mean ± SD of n = 31 observations. Differences between drinks were assessed by 1-way repeated measures ANOVA, with LSD post-hoc analyses performed to correct for multiple comparisons. *p < 0.05 compared to water, SpD1, and SpD2. ORS, oral rehydration solution; SpD, sports drink

We evaluated the difference in body mass change for each beverage. The change was significantly smaller for ORS than for water, SpD1, or SpD2 (Fig. 1).

### Cumulative urine output

We evaluated the difference in cumulative urine output for each beverage at different times. At 1 h after beverage consumption, urine output was significantly lower for ORS than for water. At 2 h after beverage consumption, cumulative urine output was lower for ORS, SpD1, and SpD2 than for water, and it was lower for ORS than for SpD2. At 3 h after beverage consumption, cumulative urine output was lower for ORS, SpD1, and SpD2 than for water. At 4 h after beverage consumption, cumulative urine output was lower for ORS, SpD1, and SpD2 than for water, and it was lower for ORS than for SpD2 (Table 4).

**Table 4:** Cumulative urine output following the consumption of test drinks at different testing times

Time	Water	ORS	SpD1	SpD2	*p*	Post-hoc
After 1 h	638 ± 245.3	543 ± 201.7	486 ± 170.8	557 ± 164.8	0.008	C < A
After 2 h	959 ± 271.7	791 ± 217.5	771 ± 209.0	830 ± 183.5	0.001	B, C, D < A, B < D
After 3 h	1110 ± 285.0	893 ± 228.9	928 ± 230.0	978 ± 201.2	0.000	B, C, D < A
After 4 h	1189 ± 293.6	956 ± 230.6	1001 ± 230.3	1057 ± 215.2	0.000	B, C, D < A, B < D

A: Water; B: ORS; C: SpD1; D: SpD2; Values are mean ± SD of n = 31 observations. Differences between drinks were assessed by 1-way repeated measures ANOVA, with LSD post-hoc analyses performed to correct for multiple comparisons (*p* < 0.05)

ORS, oral rehydration solution; SpD, sports drink

### Beverage hydration index

**Fig. 2:**
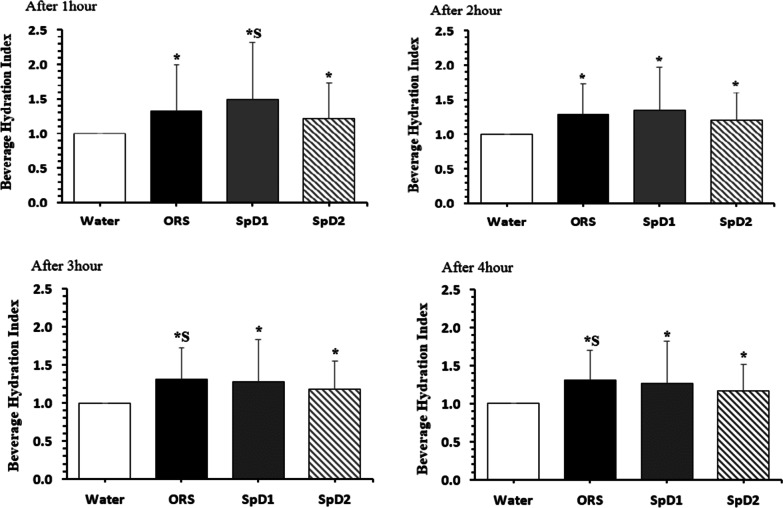
Beverage hydration index following the consumption of test drinks at different testing times. Values are mean ± SD of n = 31 observations. Differences between drinks were assessed by 1-way repeated measures ANOVA, with LSD post-hoc analyses performed to correct for multiple comparisons. *p < 0.05 compared to water; ^S^*p* < 0.05 compared to SpD2. ORS, oral rehydration solution; SpD, sports drink

We evaluated the difference in BHI (g) for each beverage at different times. At 1 h after beverage consumption, BHI was significantly higher for ORS than for water, and it was higher for SpD1 than for SpD2. At 2 h after beverage consumption, BHI was significantly higher for ORS, SpD1, and SpD2 than for water. At 3 and 4 h after beverage consumption, BHI was significantly higher for ORS, SpD1, and SpD2 than for water, and it was higher for ORS than for SpD2 (Fig. 2).

### Plasma volume change

We evaluated the difference in PVC (%) for each beverage at different times. There was no statistically significant difference in PVC for each beverage at all times of testing (Table 5).

**Table 5:** PVC following the consumption of test drinks at different testing times

Time	Water	ORS	SpD1	SpD2	*p*
After 1 h	− 0.301 ± 1.438	− 0.254 ± 1.385	− 0.463 ± 1.265	− 0.189 ± 1.265	0.852
After 2 h	− 1.430 ± 2.340	− 0.936 ± 0.928	− 0.652 ± 0.870	− 0.925 ± 1.363	0.243
After 3 h	− 1.235 ± 1.789	− 0.836 ± 1.097	− 0.896 ± 1.266	− 0.552 ± 1.747	0.343

Values are mean ± SD of n = 31 observations. Differences between drinks were assessed by 1-way repeated measures ANOVA, with LSD post-hoc analyses performed to correct for multiple comparisons

ORS, oral rehydration solution; SpD, sports drink

## Discussion

The present study compares different hydration effects from consumption of water, sports drinks, and ORS. We examined water absorption and plasma volume change in athletes based on the measurements of body weight change, cumulative urine output, BHI, and PVC.

Body weight change was significantly smaller for ORS than for water, SpD1, and SpD2, indicating that bodily water loss is smaller following the consumption of ORS than SpD1, and SpD, and water. Cumulative urine output differed only slightly in testing time, although cumulative urine output in 4 h was lower for ORS, SpD1, and SpD2 than for water, and it was lower for ORS than for SpD2. Thus, we believe that bodily water loss is smaller for ORS than for water and SpD2. According to a study by Millard-Stafford et al. [14], the cumulative urine output from 1 to 4 h after water intake was greater than that of drinks containing carbohydrates and electrolytes, and at 3 and 4 h, there is more water than a drink containing dipeptide and electrolyte, which supports the fact that the sports drink show in this study produced less cumulative urine than water.

The BHI is the urine output after consumption of water divided by the urine output after beverage consumption, and it is an index commonly used to verify the hydration effect of a beverage [13, 18]. In this study, although BHI was slightly different for each time period, ORS, SpD1, and SpD2 were higher than water from 1 to 4 h after water intake.According to Bechke et al. [2], BHI was higher in sports drinks than water at 3 and 4 h after water intake, and in a study by Millard-Stafford et al. [14], BHI was high in drinks containing carbohydrate and electrolyte and drinks containing dipeptide and electrolyte after 4 h after water intake, which supports the result of this study. On the other hand, Sollanek et al. [18] reported that ORS showed higher BHI than water, but sports drinks did not have a statistically significant difference. This means that when comparing the hydration effects of water, sports drinks, and ORS, ORS can have the greatest effect. Also, in this study, ORS showed higher BHI than SpD2 at 3 and 4 h after water intake, which is consistent with previous studies showing that sports drinks with low osmolality had a greater hydration effect [16]. Clarke et al. [3] found that ORS with a high Na+ content showed high BHI values, and this study also confirmed that ORS with a relatively high Na+ content produced high BHI values and BHI value was high in drinks with less cumulative urine output. In other words, it was confirmed that the cumulative urine output and the BHI result were inversely proportional, which could re-prove the study [13, 18] that verified that BHI is a useful index for identifying the hydration effect.

Our study compared PVC to verify the hydration effects of different beverages, but we found no significant difference. According to Clarke et al. [3], PVC showed a rapid increase immediately after beverage consumption (0–15 min) and then plateaued after 1 h. We measured PVC at ≥ 1 h after beverage consumption, which may have been responsible for the insignificant difference. Furthermore, the coefficient of variation (CV) of PVC was 2.35, which is relatively high, possibly preventing the detection of smaller differences in PVC. For participants, indicating that PVC should be an index with higher sensitivity to an individual’s physical characteristics.

Studies that have verified the hydration effects of beverages on athletes have been steadily conducted. As a result of conducting Systematic Meta-Analysis of 28 studies on the hydration effects of sports drinks and water which Rowlands et al. [16] reported, when comparing ingested hypertonic (> 300 mOsmol kg^−1^), isotonic (275–300 mOsmol kg^−1^) and hypotonic (< 275 mOsmol kg^−1^) drinks, it is reported that hypotonic drinks have the greatest effect. With the classification criteria for beverages in this study, SpD1 corresponds to isotonic drinks, SpD2 corresponds to ingested hypertonic drinks, and ORS corresponds to hypotonic drinks. Therefore, it supports the result that ORS, the main result of this study, is more effective for hydration than SpD2.

The results of this study are significant in the point that it tried to verify the hydration effect of ORS in athletes. Previous studies that tried to confirm the hydration effect of athletes have been primarily studies using sports drinks. Therefore, the results of this study may suggest ORS as a drink that can help improve athletes’ performance along with sports drink. Nevertheless, this study has the following limitations. First, participants in this study were limited to athletes in throwing, bowling, and gymnastics events, so it is not easy to broadly interpret the effects of ORS for athletes in all sports. It is expected that a follow-up study will verify the effect of ORS in athletes in various sports. Second, in the case of female athletes, it was judged that external factors could be included in the study results due to their physical characteristics (menstruation), so this study selected male athletes as the study subjects. Thus, there is a need to verify the effectiveness of ORS in female athletes in follow-up studies. Third, the study subjects who participated in this study were university students, so ORS effect may be different for younger athletes. Therefore, it is expected that a follow-up study will verify the effectiveness of ORS in various age groups.

## Conclusions

In conclusion, our study evaluated the hydration effects of beverages, such as water, sports drinks, and ORS, on athletes. The ORS and sports drinks were more effective for hydration than water, and the difference between ORS and sports drinks varied depending on the type of sports drink.

## Data Availability

The datasets used and/or analysed during the current study are available from the corresponding author on reasonable request.

## Abbreviations

ACSM: American College of Sports Medicine
BHI: Beverage hydration index
CV: Coefficient of variation
ES: Effect size
ORS: Oral rehydration syrup
PVC: Plasma volume change
SPSS: Statistical package for social sciences
SpD: Sports drink
WHO: World Health Organization
